# Benthic community structure on coral reefs exposed to intensive recreational snorkeling

**DOI:** 10.1371/journal.pone.0184175

**Published:** 2017-09-05

**Authors:** Bobbie Renfro, Nanette E. Chadwick

**Affiliations:** Department of Biological Sciences, 101 Rouse Life Sciences Building, Auburn University, Auburn, Alabama, United States of America; Academia Sinica, TAIWAN

## Abstract

Chronic anthropogenic disturbances on coral reefs in the form of overfishing and pollution can shift benthic community composition away from stony corals and toward macroalgae. The use of reefs for recreational snorkeling and diving potentially can lead to similar ecological impacts if not well-managed, but impacts of snorkeling on benthic organisms are not well understood. We quantified variation in benthic community structure along a gradient of snorkeling frequency in an intensively-visited portion of the Mesoamerican Barrier Reef. We determined rates of snorkeling in 6 water sections and rates of beach visitation in 4 adjacent land sections at Akumal Bay, Mexico. For each in-water section at 1–3 m depth, we also assessed the percent cover of benthic organisms including taxa of stony corals and macroalgae. Rates of recreational snorkeling varied from low in the southwestern to very high (>1000 snorkelers d^-1^) in the northeastern sections of the bay. Stony coral cover decreased and macroalgal cover increased significantly with levels of snorkeling, while trends varied among taxa for other organisms such as gorgonians, fire corals, and sea urchins. We conclude that benthic organisms appear to exhibit taxon-specific variation with levels of recreational snorkeling. To prevent further degradation, we recommend limitation of snorkeler visitation rates, coupled with visitor education and in-water guides to reduce reef-damaging behaviors by snorkelers in high-use areas. These types of management activities, integrated with reef monitoring and subsequent readjustment of management, have the potential to reverse the damage potentially inflicted on coral reefs by the expansion of reef-based recreational snorkeling.

## Introduction

Stony corals compete both among themselves and with other non-reef-building organisms for dominance on tropical coral reefs [1]. Natural disturbances on coral reefs, such as storms, may alter the species composition of benthic organisms, but usually do not shift the system away from reef-builders [2,3]. In contrast, chronic anthropogenic disturbances including pollution and overfishing can overwhelm the system and reduce the abundance of reef-building species [2,4]. Macroalgae are major competitors for space with stony corals on tropical reefs [5,6]; they are prevented from becoming dominant through the activities of grazing fishes and sea urchins [7], and the effects of naturally low levels of dissolved nutrients which limit their growth [8,9]. Dominance on coral reefs can shift toward algae and away from stony corals through localized human activities such as overharvesting of herbivorous fishes, dumping of sewage, and excessive use of fertilizers along adjacent coastlines, as well as large-scale processes including effects of climate change and regional sea urchin die-offs [10–15]. High rates of SCUBA diving and snorkeling may cause damage to stony corals ([16] and references therein), but their impacts on the relative dominance of stony corals versus macroalgae remain largely unknown, thus far examined in only one study [17].

Ecotourism in the form of recreational diving and snorkeling has been hailed as a solution to the ecosystem degradation caused by extractive reef uses such as fishing and mining, because it incentivizes the preservation and non-destructive use of reef resources [18]. The development of diving tourism on coral reefs is potentially an ideal avenue for some tropical countries to utilize their diverse marine resources while avoiding extractive processes that degrade this important resource base. However, sustaining a recreational diving industry requires regulation of the number of people who visit a given underwater habitat each year, and of their actions as they interact with marine ecosystems [19–21]. Divers are willing to pay more to visit healthy versus degraded coral reefs, and will avoid damaged reef areas if given the choice [22,23], providing economic incentives for communities to manage their reefs to maintain biodiversity. Snorkelers and divers who receive pre-dive educational briefings, coupled with in-water surveillance by dive guides, cause significantly less damage to coral reefs than do less well-managed visitor groups [16]. However, in many regions recreational diving on coral reefs remains unregulated and intense, causing degradation that risks collapse of both the ecosystem and the local diving industry that depends on it [17].

When snorkelers and divers accidentally contact the reef with their bodies, fins, or other diving gear, they often cause damage by fracturing the skeletons and abrading the thin tissues of living stony corals [16,24]. This physical damage weakens stony corals, making them more susceptible to predators and diseases [25–27] that eventually may cause coral death and clear reef space for macroalgal colonization [5,28]. The presence of snorkelers on coral reefs also causes herbivorous fishes to exhibit escape behaviors, thereby reducing fish abundance on reefs [17], as well as interfering with their grazing behavior [29]. Herbivores play a major role on reefs by consuming macroalgae and reducing the ability of algae to compete for space with stony corals [30]. Reefs that experience a severe reduction in herbivory may become dominated by macroalgae rather than reef-building stony corals [6,31].

Reduction in the abundance of herbivorous fishes also may lead to an increase in the abundance of sea urchins through competitive release, because both groups consume benthic algae [32]. Sea urchins serve an important role as major reef herbivores [32,33]. Reefs where either herbivorous fishes or urchins have been removed by overfishing or disease may be able to maintain stony coral dominance due to the presence of the redundant herbivore group, but these systems are likely to collapse when both groups are removed [31]. Urchins impact the reef in complex ways; they play a role in stony coral degradation by scraping both juvenile stony corals and macroalgae from reefs, leading to potentially rapid bioerosion of reef substrate [34]. High abundances of sea urchins thus can cause damage to reef ecosystems [34,35]. Consequently, sea urchins may influence reef resilience either positively or negatively depending on their abundance relative to reef fishes, including the wrasses and triggerfishes which consume them [36,37]. Assessment of sea urchin abundance as a component of the benthic community therefore is needed to understand the complex cascade effects of human disturbances on coral reefs.

**Fig 1:**
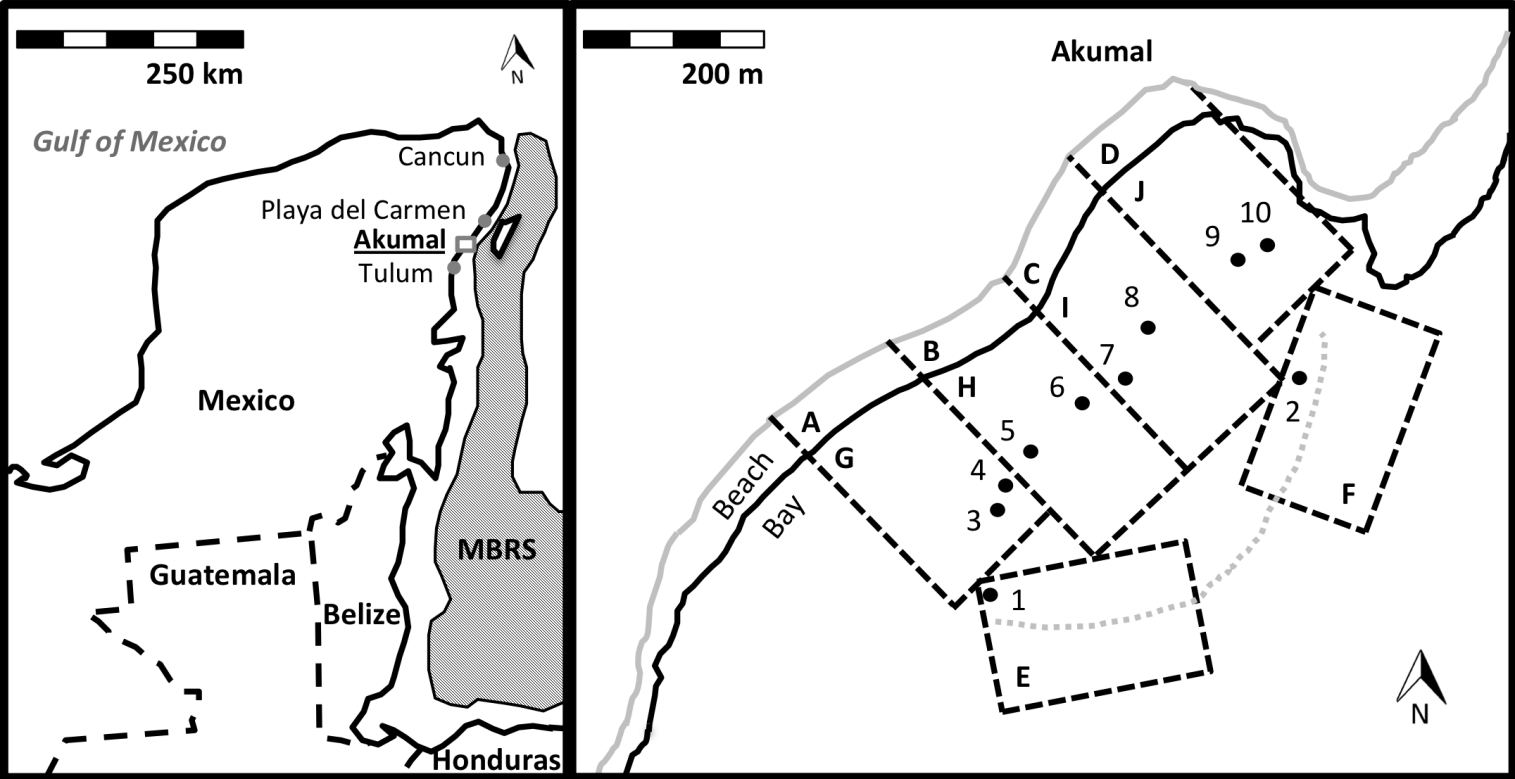
**Maps of (a) Yucatan Peninsula showing major tourist cities, and (b) Akumal Bay showing areas sampled in the present study.** In (a), the extent of the Mesoamerican Barrier Reef System (MBRS) is shown as a shaded area along the coastlines of 4 countries: Mexico, Belize, Guatemala, and Honduras. The study site at Akumal is underlined and marked with a rectangle to indicate the area shown in detail in (b). The detail map in (b) presents the 10 bay sections in which visitor abundances were quantified, each identified by letter in the upper left corner of the section (delineated by dashed lines): 4 sections on land along the beach, sampled for beach visitors (A-D), and 6 in the water, sampled for snorkeling visitors (E-J). G to J are inner bay sections that parallel the on-land sections A to D, and E and F are reef crest sections along the mouth of the outer bay. Shown also are patch reefs #1–10 sampled for coral reef benthic community composition: 2 reefs in each of the 4 inner bay sections, and 1 reef each in the 2 outer bay sections. The landward edge of the sandy beach is delineated by a solid grey line, the water’s edge (coastline) by a solid black line, and the coral reef crest by a dotted line. For details and site comparisons see text here and maps in [17,43,46].

The Mesoamerican Barrier Reef System (MBRS, [38]) is the second largest contiguous coral reef tract in the world, spanning much of the western edge of the Caribbean Sea along the coastlines of southeastern Mexico, Belize, Guatemala and Honduras (Fig 1A). Coastal tourism associated with the MBRS has been rising over the past several decades in Mexico, beginning with the conception of Cancun as a pre-planned tropical tourism mecca [38,39]. Continuing growth associated with the tourism industry has expanded the human population in the region > 15-fold in 40 y, to ~ 1.5 million in 2013 [40,41], mostly in the cities of Cancun and Playa del Carmen [42,43]. The tourism industry recently has expanded down the coastline toward less congested towns such as Akumal (~ 105 km south of Cancun), which is experiencing rapid growth due to resident sea turtle populations that have attracted busloads of snorkelers [17]. Coral reefs in the most intensively-visited portions of Akumal Bay are beginning to exhibit signs of degradation [17], but patterns of alteration in their benthic community structure remain mostly unquantified. Information is needed to provide baseline values for tracking potential changes in the structure of these reefs as rates of visitation continue to change, especially in low-use areas of the bay that have not been visited frequently. Akumal Bay is a major feeding and nesting site for green and loggerhead sea turtles, which frequent both seagrass beds and patch reefs in the bay [43,44]. In 2016, Akumal Bay was declared a refuge for protected marine species by PROFEPA (Federal Attorney for Environmental Protection under the Mexican Government [45]). The reefs of this bay thus represent a model system for assessment of variation in coral reef condition with levels of recreational snorkeling, as well as for the evaluation of sustainable reef management practices.

Here we quantify variation in the benthic community structure of patch reefs in Akumal Bay with levels of in-water recreational snorkeling and on-land beach use by visitors. We analyze patterns of space use exhibited by stony corals, macroalgae, and other benthic organisms in relation to levels of snorkeling. We then discuss the implications of these patterns for the sustainable management of recreational snorkeling on coral reefs.

## Materials and methods

### Study sites

The present study was conducted during May to July 2015 on coral reefs in Akumal Bay, Quintana Roo, Mexico (20°24’00”N, 87°19’16”W, Fig 1). This bay contained shallow coral patch reefs that were interspersed with sea grass beds, and bordered by a fairly continuous reef crest along the seaward margin [17,44,46,47]. Benthic sampling for the present study focused on the patch reefs and did not examine the sea grasses (phanerogams) in this bay. The reef crest protected the bay from high levels of water motion, creating a shallow inner area that usually exhibited calm water conditions and attracted large numbers of recreational snorkelers [44]. SCUBA diving was prohibited inside the bay (Centro Ecologico Akumal [CEA], http://ceakumal.org/). CEA issued the permission for access to the study locations. These field studies did not involve contact with any endangered or protected species.

Most tourism infrastructure and the main street of Akumal were located along the northeastern bay; most visitors thus entered the beach in this area and remained in the north bay. This created a gradient of both on-land beach use and in-water snorkeling activity, from high in the northeast (~357 visitors in the water and on the beach within ~ 300 x 300 m area during maximum use at 15:00 h each day in the High Use zone, as measured in 2013) to low in the southwest bay (< 65 visitors in the water and on the beach within ~ 300 x 300 m area at 15:00 h, in the Low Use zone; CEA; [17]). Based on these data and our preliminary observations, we partitioned the on-land beach area into 4 sections of approximately equal distance along the shoreline (sections A-D), and the in-water area into 4 adjacent inner bay sections containing patch reefs plus 2 outer bay sections along the continuous reef crest (sections E-J, Fig 1B), for quantification of variation in visitation levels. We included the 2 outer bay sections because similar to the inner bay sections, they also contained patch reefs and preliminary observations indicated that they received limited visitation, even though they differed in physical conditions from the 4 inner bay sections. Statistical analyses of trends were conducted among all 6 sections, and also among only the 4 inner bay sections (see below). Each of the 4 on-land sections was ~150 x 50 m area, and each of the 6 in-water sections was ~150 x 250 m area; some extended further out to sea than did others, due to curvature of the shoreline and reef crest (Fig 1B). To provide enhanced information on patterns of visitor use, the previous High and Low Use zones [17] each were divided into 2 inner bay sections (I, J, and G, H, respectively), and we added 2 outer bay sections. These 6 in-water sections corresponded to 15 even more finely-divided quadrats examined in a separate study for sea anemone and crustacean abundances [46]: Section E corresponded to Quadrats 14 and 15; Section F to Quadrat 13; Section G to Quadrats 5, 6, 11, and 12; Section H to Quadrats 3, 4, 8, and 9; Section I to Quadrats 2 and 8; and Section J to Quadrats 1 and 7 (compare Fig 1B with map in [46]).

Within each of the 4 inner bay water sections (G, H, I, J), we selected 2 patch reefs to quantify benthic community structure. Patch reefs were selected based on their similarity in size (~150 m^2^ area), depth (1.3–1.7 m deep), and distance from shore (~ 120–200 m distant), to ensure that reefs exposed to roughly similar physical conditions were sampled within each section (reefs #3–10, Fig 1B). We also selected 1 patch reef in each of the 2 outer bay reef crest sections (E and F) that were similar to each other in size (~ 150 m^2^ area), depth (2.5–3.0 m deep), and distance from shore (~ 250–300 m distant, reefs #1–2 in Fig 1B; data from inner vs. outer bay sites were analyzed together and separately, see Data Analyses). Only 1 reef site per outer bay section was selected, near the edge of each section because few live patch reefs occurred near the reef crest due to previous damage from hurricanes Emily and Wilma [48,49]. Reef sites (#1–10), beach sections (A-D), and bay sections (E-J) each were numbered (or alphabetized) within each group from relatively low to high levels of visitor use, based on the data collected below.

### Patterns of visitor abundance

We defined visitors as all people occurring within a given section of land or water during each observation period. We recorded patterns of visitor abundance in the 4 beach and 6 bay sections during 7 periods each day for 5 d, to allow spatial comparison of visitation rates between these 2 areas. Five dates for conducting visitor abundance surveys (10, 12, 26, 29, and 30 July, 2015) were selected because they were sunny (rather than rainy which deterred people from visiting the bay), and also were dates during which the beach contained relatively low levels of washed-up macroalgae *Sargassum* sp. [50], which on other days partially obstructed visitor entry into the water. As such, visitor abundances reported here likely represent maximal daily values during summer 2015. Our preliminary observations indicated that spatial patterns of visitor use quantified using this method were representative of similar patterns revealed by long-term visitor monitoring at this site [17].

On each of the 5 selected dates, 2 observers (B. Renfro and a field assistant) walked the entire length of the beach area (from section D to A) at the start of every other daylight hour between sunrise and sunset (06:00, 08:00, 10:00, 12:00, 14:00, 16:00, and 18:00; N = 7 periods d^-1^, for the first 10–20 min of each 2-h period). One observer counted all visitors on each section of the beach, while the other simultaneously counted all visitors (wading, floating, swimming, or snorkeling) in the adjacent inner bay water section, and in the 2 outer bay water sections. The coordinated counting of visitors on land and in the water prevented double counting of any who moved between sections during a given period. It was difficult to identify the presence of a snorkel on some in-water visitors, especially those located in the outer bay near the reef crest. However, most persons observed in the water appeared to use a mask and snorkel and thus to function as snorkelers (rather than as waders or swimmers), so here are referred to interchangeably as snorkelers or as in-water visitors.

### Coral reef community structure

We quantified benthic community composition on the 10 selected patch reefs by deploying 10 line transects parallel to shore on each reef. Each transect was 10–15 m long depending on patch reef shape, and was deployed parallel to the other transects ~1 m apart (methods adapted from [51]), beginning at the near-shore edge of the reef. Along each transect, 3 points were selected using a random number generator, then at each point, a 1-m^2^ quadrat gridded with string to contain 100 10-cm^2^ cells was placed carefully on the reef surface. The identity of the substrate that occupied the most area within each cell was recorded for each of 4 major categories (stony corals, macroalgae, other sessile organisms, and non-living substrate) within 3 quadrats per transect x 10 transects per reef (after [16,52,53]). Stony corals were defined as scleractinian reef-building corals, and were identified to species as: *Agaricia agaricites*, *A*. *tenuifolia*, *Diploria clivosa*, *D*. *strigosa*, *Montastraea cavernosa*, *Orbicella annularis*, *O*. *faveolata*, *Porites astreoides*, *P*. *divaricata*, *P*. *furcata*, *P*. *porites*, *Siderastrea siderea*, *Acropora cervicornis*, or *A*. *palmata* (the 14 most common stony coral species in Akumal Bay, as indicated by preliminary observations), or grouped as other stony coral species (identified using [54]). Macroalgae were identified as belonging to 4 growth forms: crustose coralline algae (CCA), erect calcified macroalgae (CMA), erect fleshy macroalgae (FMA), or turf algae [55]. Other sessile organisms were classified in 5 groups: gorgonians (including soft corals and sea fans), fire corals (defined as hydrocorals in the genus *Millepora*), sponges, sea anemones, or zoanthids (zoantharians) [55,56]. No other organisms occupied the majority of any 10-cm^2^ cell surveyed, see above. Non-living substrate was recorded in 3 categories: sand, rubble, or dead stony coral not colonized by turf algae. We examined the cells within each quadrat from directly overhead, so that horizontal percent cover was estimated for all categories, including for organisms that also extended vertically into the water column, such as some stony and soft corals and sea fans.

Within each sampled quadrat, we also assessed the abundances of sea urchins (long-spine sea urchins *Diadema antillarum*, and other less-common sea urchins grouped together, after [57]). If more than half the body of a sea urchin occurred within a given quadrat, it was counted as present [33]. Reef crevices below the quadrat were examined thoroughly to detect the presence of sea urchins.

### Data analyses

We analyzed visitor abundances by focusing on the peak-use periods of 14:00 h on the beach and 16:00 h in the water (see Results), to reveal patterns during the maximal-use periods each day. Visitor abundances were not normally distributed among days (Q-Q Plots, Shapiro-Wilk normality test), so we employed a Generalized Linear Model (GLM) assuming a Poisson distribution for count data in R version 3.1.3 [58], to determine variation in visitor abundance among sections of the bay. The Poisson regression assumption of equidispersion was verified using the dispersion test in R package AER. In cases where over-dispersion was found, the model was re-fit with quasipoisson distribution to account for the data dispersion pattern. Post-hoc comparisons among bay sections were made using Tukey’s Honest Significant Difference (HSD) test, with R function Simultaneous Tests for General Linear Hypotheses.

To assess patterns of benthic community structure, we calculated the mean percent cover of each benthic category within each of the 10 transects examined per reef, by averaging values from the 3 quadrats examined per transect, to yield 10 benthic samples per reef. We ranked the 10 reefs from low to high in terms of their exposure to snorkelers in each water section of the bay, based on the snorkeler count data (see above), and the location of each reef within each section. The northern patch reef within each section received more snorkelers than did the southern reef, because it was closer to the adjacent section where higher relative snorkeler levels were observed. This ranking of reef sites allowed more fine-scale spatial analysis than did use of the absolute snorkeler count data, which were recorded only for each large section of the bay (Fig 1B). Benthic cover in each category for each sample was regressed on the ranked snorkeling level for each reef site, by fitting an ordinary least squares regression (OLS) in R. We also regressed benthic cover on the ranked snorkeling levels of only the 8 inner bay sites (ie. excluding the 2 outer bay sites #1–2, see Fig 1), to examine variation among only the inner bay sites, which all occurred at the same water depth (see above). Residuals were examined using normal Q-Q plots and Shapiro-Wilk normality test. Data were transformed where necessary to fit model assumptions. Raw data are presented in a Supporting Information file (S1 Table. All results are presented as means ± standard error unless otherwise noted.

## Results

### Patterns of visitor abundance

**Fig 2:**
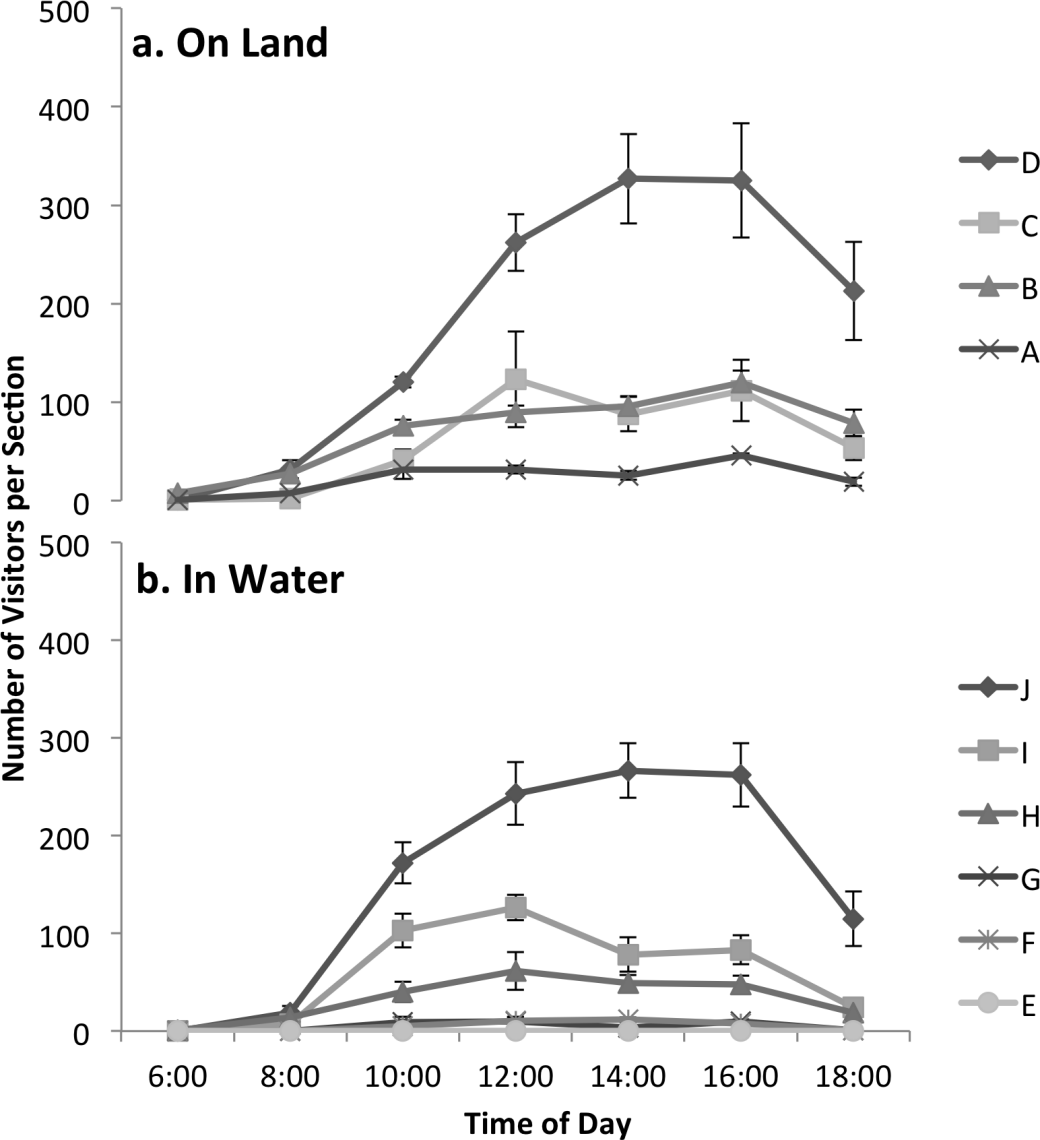
Spatial and temporal variation in visitor abundance at Akumal Bay, Mexico, during July 2015. Shown are numbers of visitors ($\bar{x}$ ± SE, N = 5 d) observed every 2 h between sunrise (06:00) and near sunset (18:00) in: (a) 4 on-land beach sections, and (b) 6 in-water sections (also referred to as snorkelers). See map (Fig 1) and text for details.

During the morning hours on all observed days, visitor abundance was low both on land and in the water. Visitor abundance increased toward afternoon to a daily peak at 14:00 h on the beach and 16:00 h in the water, when 536 ± 74 and 414 ± 44 visitors were observed on land (all beach sections combined) and in the water (all in-water sections combined), respectively, and then declined sharply toward sunset (Fig 2).

During peak beach use at 14:00 h, visitor levels varied significantly among on-land sections of the bay (GLM, p < 0.00l). On-land visitor occupancy in the northeastern section D, where the public accessed the beach from the main road in Akumal, was significantly higher than in all 3 other beach sections (p < 0.001 for pairwise comparisons, Table 1A). In contrast, sections C and B, where the beach transitioned from a public to a private area, exhibited moderate visitor abundances that did not significantly differ from each other (p = 0.98, Table 1A). Section A, which was a private beach for a large resort hotel in the southwest end of the bay, possessed significantly lower visitor abundances than did the other 3 beach sections (p < 0.01 for pairwise comparisons, Table 1A).

**Table 1:** Results of simultaneous tests for general linear hypotheses of variation in visitor abundance among sections of Akumal Bay, Mexico. Shown are Z and P values respectively for post-hoc pairwise comparisons between bay sections: (a) on land during peak use at 14:00 each day in 4 sections (A-D), and (b) in the water during peak use at 16:00 each day in 6 sections (E-J).

(a) On-land		A	B	C	-	-
B	Z	3.481				
	P	< 0.01				
C	Z	3.220	-0.346			
	P	< 0.01	0.98			
D	Z	7.282	6.198	6.417		
	P	<0.001	<0.001	<0.001		
(b) In-water		E	F	G	H	I
F	Z	1.263				
	P	0.77				
G	Z	1.533	0.363			
	P	0.59	0.99			
H	Z	3.496	3.622	3.496		
	P	< 0.01	< 0.01	< 0.01		
I	Z	4.181	4.884	4.887	2.343	
	P	< 0.001	< 0.001	< 0.001	0.14	
J	Z	5.588	7.506	7.827	8.349	7.042
	P	< 0.001	< 0.001	< 0.001	< 0.001	< 0.001

Abundances of visitors in the water (snorkelers) were ~ 23% lower than those on the beach at all times of day, and varied significantly among in-water sections (GLM, p < 0.00l). In the most frequently-visited northeastern section J, 262 ± 33 snorkelers were observed in the water during peak use at 16:00 h, with > 1,000 snorkelers visiting this area over the course of an entire day (Fig 2B). Snorkeler abundance in section J was significantly higher than in all other in-water sections (p < 0.001 for all pair-wise comparisons, Table 1B). In contrast, mid sections of the inner bay (I and H) received relatively moderate numbers of snorkelers that differed significantly from all other in-water sections, but not from each other (p = 0.14, Table 1B). The southern and outer bay sections (E, F, and G) contained low in-water snorkeler abundances that differed from those in all other sections, but not significantly from each other (p = 0.59–0.99 for pairwise comparisons, Table 1B; < 10 snorkelers in the water at 16:00, for a total of < 36 snorkelers d^-1^). Overall, in-water snorkeler abundances appeared to vary strongly with time of day, distance from shore, and distance along the shore from the main public access point in the northeastern bay.

### Coral reef community structure

**Fig 3:**
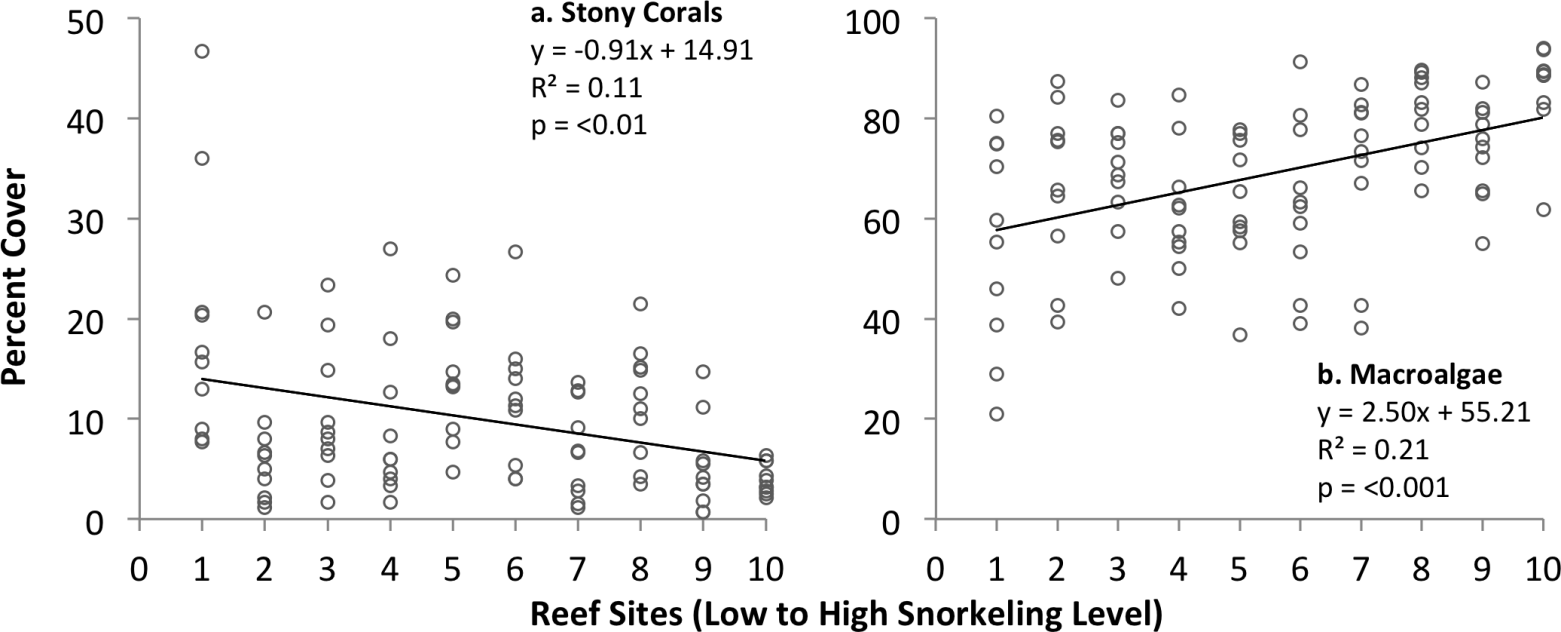
Variation in the percent cover of stony corals (left) and macroalgae (right) among patch reefs ranked by level of snorkeling in Akumal Bay, Mexico. N = 10 transects examined per reef.

The percent cover of live stony corals on patch reefs was low (4.0–19.4%, range of mean values per reef), while macroalgal cover was ~ 2-20x higher (55.0–85.9%). Percent cover ranged 4.0–17.2% for other benthic organisms, and 6.0–20.8% for non-living substrates (N = 10 patch reefs). Live stony coral cover decreased significantly with snorkeler abundance; in contrast, macroalgal cover showed the inverse pattern of significant increase with snorkeler abundance on the reefs (Fig 3). Both trends remained significant when examining variation among only reefs in the inner bay, which all occurred at the same water depth (see Methods), and the R^2^ values of the relationships increased (Table 2).

**Table 2:** Results of ordinary least squares regression (OLS) analysis of variation in the percent cover of benthic organisms among patch reefs ranked by level of snorkeling in Akumal Bay, Mexico. All categories of organisms were quantified as percent cover except for sea urchins, which were assessed as abundance of individuals. Seagrasses (phanerogams) did not occur on the patch reefs and were not included in the present table. Columns include values for all 10 patch reefs examined in the bay, except for the last 2 columns which show values for only the 8 inner bay patch reefs, excluding the 2 outer bay patch reefs (see map Fig 1B for reef locations). The percent cover of both *M*. *cavernosa* and *A*. *palmata* was too low to be analyzed statistically for variation among only the 8 inner bay sites. Mounding, branching, and plating corals were defined as all species belonging to each of these 3 major growth forms of stony corals. ^ Data log_10_-transformed, † Data square root-transformed. See text for details.

Type of benthic organism	SE	T	R^2^	P	R^2^ inner bay only	P inner bay only
Live stony corals^	0.013	3.244	0.097	< 0.01	0.111	< 0.01
Mounding corals^†^	0.038	0.399	0.002	0.69	0.012	0.33
Branching corals^†^	0.034	-3.044	0.086	< 0.01	0.061	< 0.05
Plating corals^†^	0.034	-3.025	0.085	< 0.01	0.092	< 0.01
*A*. *agaricites*	0.067	-1.222	0.015	0.22	0.028	0.14
*A*. *tenuifolia*^*†*^	0.034	-2.847	0.076	< 0.01	0.039	0.08
*D*. *clivosa*	0.003	-0.877	0.008	0.38	0.001	0.77
*D*. *strigosa*	0.064	-4.084	0.145	< 0.001	0.035	0.10
*O*. *annularis*^*†*^	0.036	2.491	0.060	< 0.05	0.004	0.56
*O*. *faveolata*^*†*^	0.036	1.445	0.021	0.15	< 0.001	0.98
*M*. *cavernosa*	0.030	-1.818	0.033	0.07	N/A	N/A
*P*. *astreoides*^*†*^	0.021	-0.385	0.002	0.70	0.055	< 0.05
*P*. *divaricata*	0.025	-0.652	0.004	0.52	0.0001	0.93
*P*. *furcata*	0.055	-0.600	0.004	0.55	0.027	0.15
*P*. *porites*^*†*^	0.009	3.579	0.116	< 0.001	0.089	< 0.01
*S*. *siderea*	0.034	-0.581	0.003	0.56	0.009	0.41
*A*. *cervicornis*	0.039	-0.922	0.009	0.36	0.045	0.06
*A*. *palmata*	0.145	-2.838	0.076	< 0.01	N/A	N/A
Other stony corals	0.021	-0.092	0	0.93	0.0001	0.91
*Millepora* fire corals	0.049	-4.085	0.146	< 0.001	0.002	0.68
Gorgonians^†^	0.042	-2.048	0.041	< 0.05	0.097	< 0.01
Total algae	0.497	5.027	0.205	< 0.001	0.208	< 0.001
Turf algae	0.537	-9.104	0.458	< 0.001	0.205	< 0.001
Calcified macroalgae^†^	0.037	2.772	0.073	< 0.01	0.113	< 0.01
Fleshy macroalgae^†^	0.031	-3.336	0.102	< 0.01	0.099	< 0.01
Crustose coralline algae^†^	0.055	-5.776	0.254	< 0.001	0.028	0.13
Zoanthids (zoantharians)	0.019	-2.139	0.045	< 0.05	0.002	0.67
*D*. *antillarum*^†^ sea urchins	0.023	-2.739	0.071	< 0.01	0.061	< 0.05
Other sea urchins^†^	0.018	4.053	0.147	< 0.001	0.079	< 0.01

**Fig 4:**
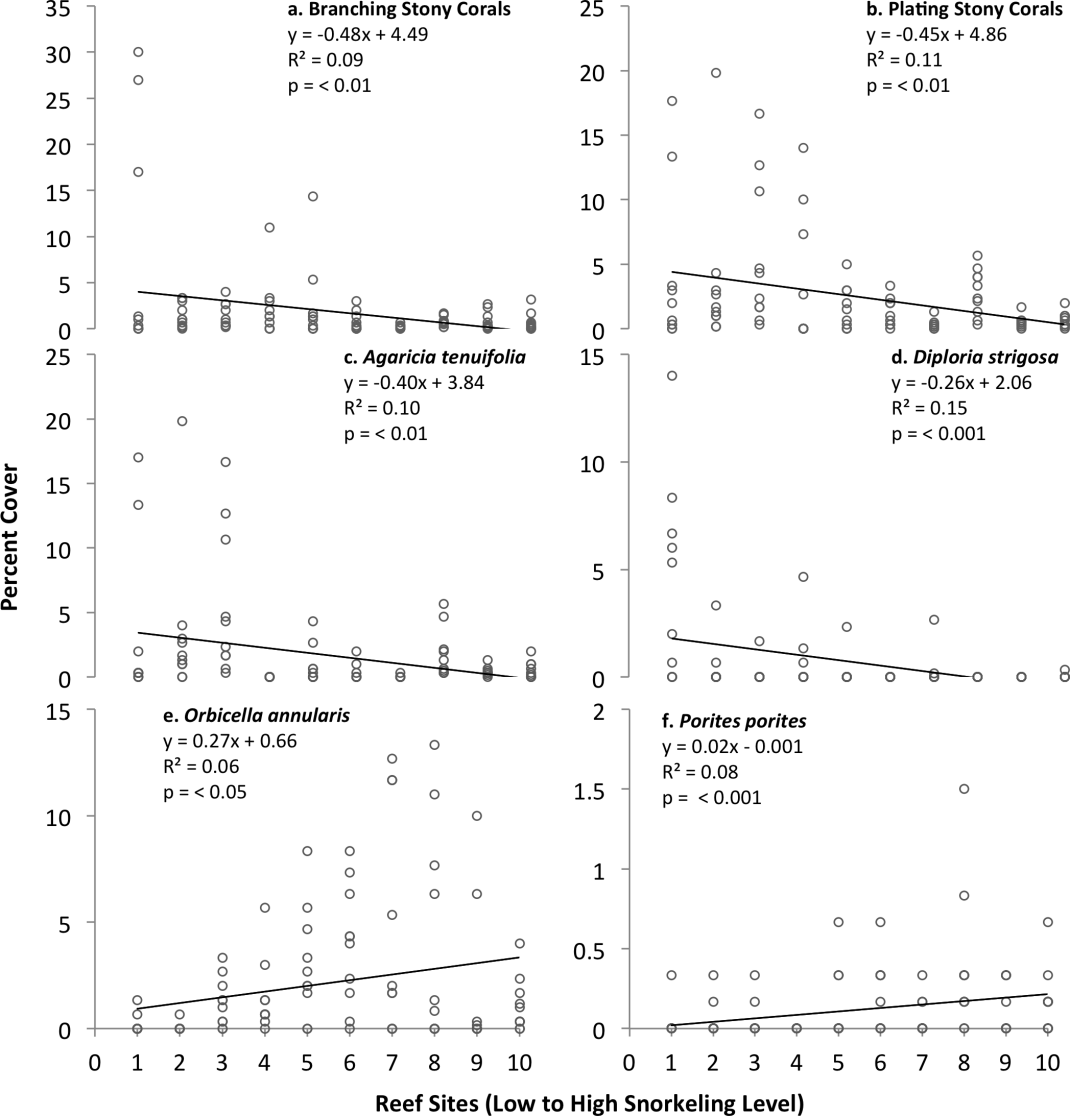
**Growth form (a,b) and species-level (c-f) variation in the percent cover of stony corals among patch reefs ranked by level of snorkeling in Akumal Bay, Mexico.** N = 10 transects examined per reef.

Stony corals with mounding growth forms occupied more space (1.5–9.9% cover) than did other types of stony corals, and did not vary significantly with snorkeler abundance. In contrast, stony corals with branching and plating (foliaceous) morphologies covered less reef area (0.21–7.67% and 0.35–5.57% cover, respectively), and both decreased significantly with levels of snorkeling (Fig 4). These trends remained the same when considering variation among only the 8 inner bay sites (Table 2). At the species level, mounding stony corals exhibited contrasting trends: colonies of the symmetrical brain coral *Diploria strigosa* decreased significantly in percent cover with snorkeling level, while boulder star corals *Orbicella annularis* increased significantly (Fig 4), however both trends became non-significant when considering only variation among inner bay sites (Table 2). In contrast, the percent cover of mustard hill corals *Porites astreoides* increased significantly with snorkeling level among the inner bay sites, but not when the outer bay sites were included.

Colonies of branching elkhorn coral *Acropora palmata* and foliaceous thin leaf lettuce coral *Agaricia tenuifolia* decreased significantly with snorkeling level, although *A*. *palmata* did so only slightly, and only when considering variation among all reef sites. Colonies of branching finger coral *Porites porites* exhibited the opposite trend (Fig 4), which remained significant among only the inner bay sites (Table 2). None of the other 9 identified stony coral species, or the other grouped stony corals, exhibited significant trends with snorkeling level.

**Fig 5:**
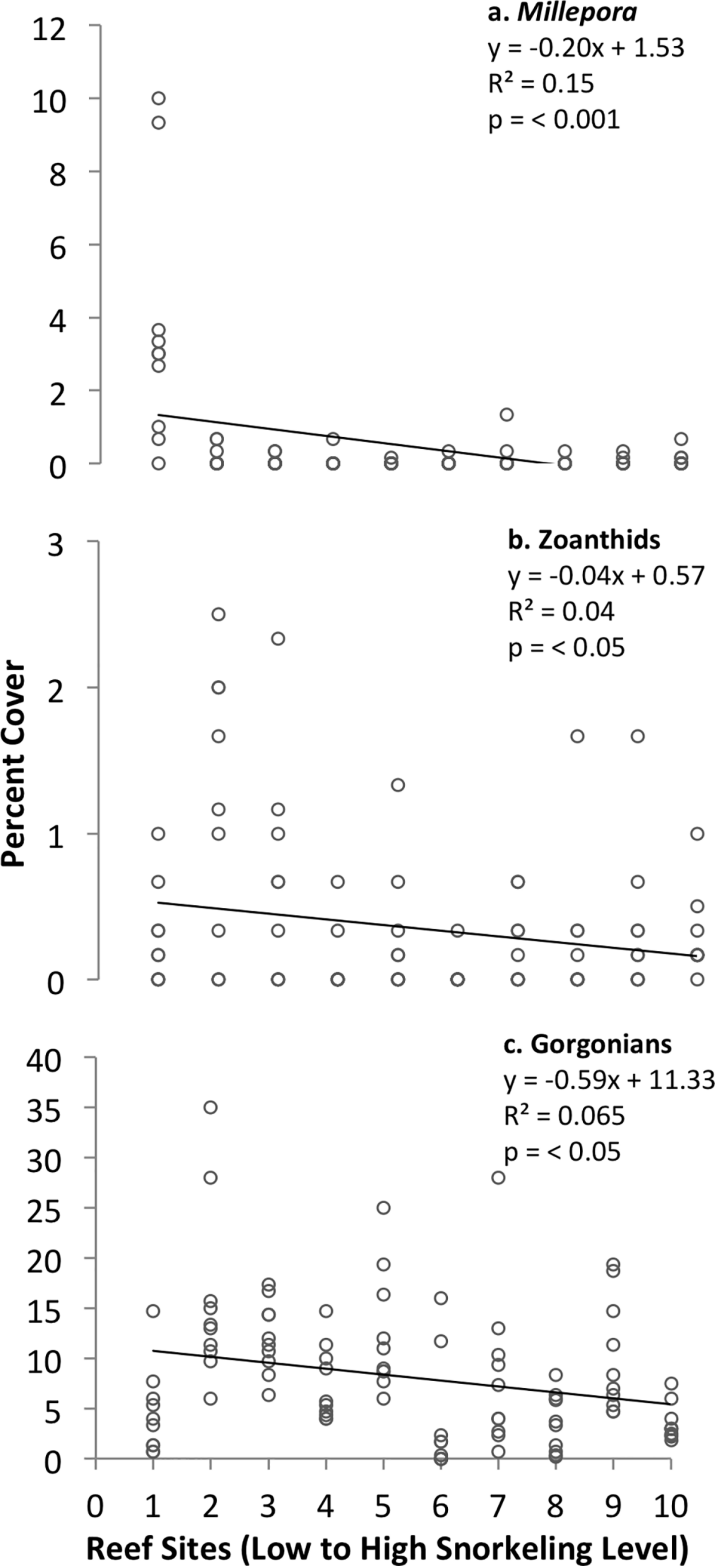
**Variation in the percent cover of non-coral sessile animals (a: *Millepora* fire corals, b: zoanthids [zoantharians], and c: gorgonians) among patch reefs ranked by level of snorkeling in Akumal Bay, Mexico.** N = 10 transects examined per reef.

Three of the 5 major types of non-coral sessile animals (zoanthids [zoantharians], *Millepora* fire corals, and gorgonians) decreased significantly with increasing snorkeling level (Fig 5), but the trend remained significant for only gorgonians among reefs in the inner bay (Table 2). Two types of benthic organisms (sea anemones and sponges) occurred at such low percent cover on the examined patch reefs, that their patterns could not be analyzed statistically.

**Fig 6:**
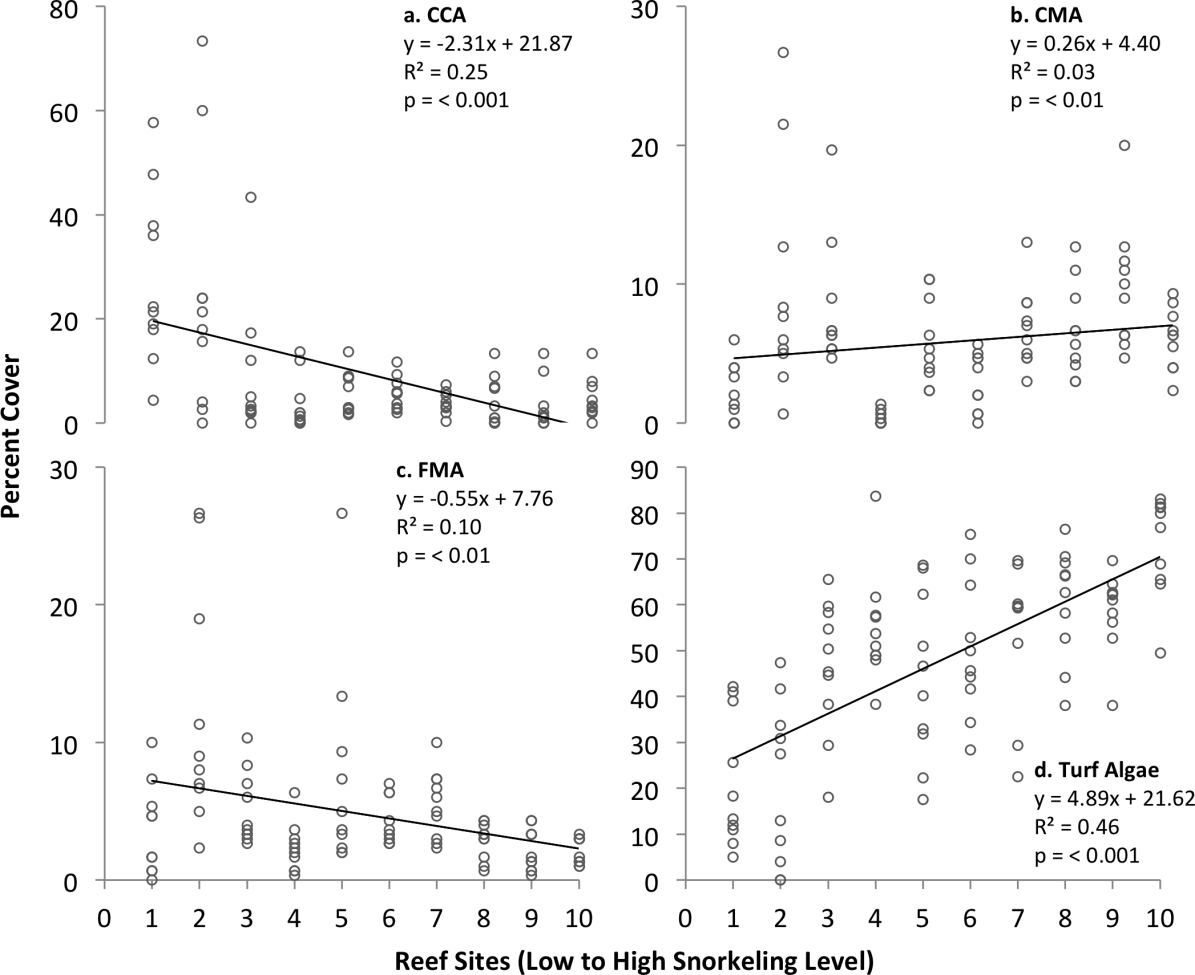
Growth form variation in the percent cover of macroalgae among patch reefs ranked by level of snorkeling in Akumal Bay, Mexico. CCA = Crustose coralline algae, CMA = Calcified macroalgae, FMA = Fleshy macroalgae. N = 10 transects examined per reef.

Turf algae were the dominant benthic organisms on many of the patch reefs examined (21.50–73.27% cover); erect calcified macroalgae (CMA, 0.33–9.73% cover) and other macroalgal types were rare. Turf algae and CMA both increased significantly in percent cover with levels of snorkeling, although CMA only slightly so. Crustose coralline algae (CCA) and erect fleshy macroalgae (FMA), while uncommon, both decreased significantly with snorkeling level (Fig 6). All but one of the macroalgal trends remained significant when considering variation among only the inner bay sites (Table 2).

**Fig 7:**
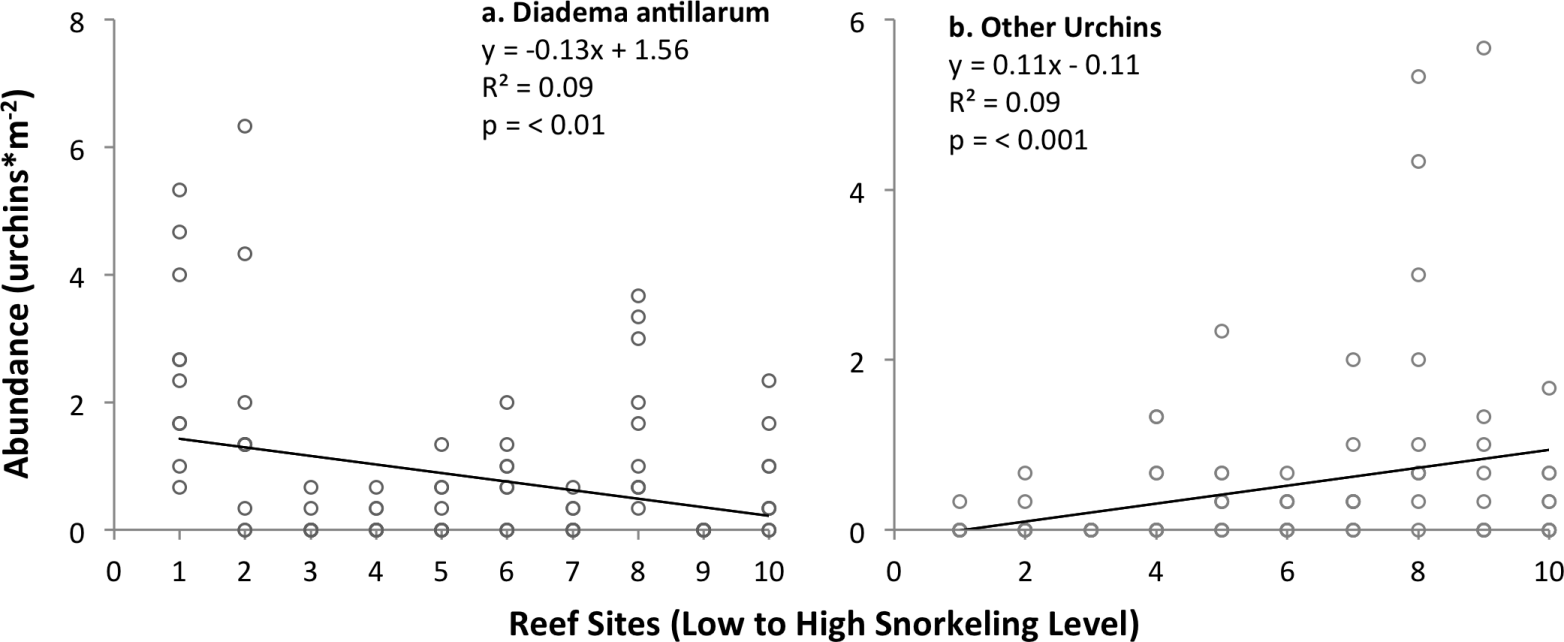
Variation in the abundance of *Diadema antillarum* and other sea urchin species grouped (*Arbacia punctulata* and *Eucidaris tribuloides*) among patch reefs ranked by level of snorkeling in Akumal Bay, Mexico. N = 10 transects examined per reef.

Long-spined sea urchins *Diadema antillarum* occurred at abundances of 0–3 individuals m^-2^ (range of mean values per reef), and were most common on the offshore patch reefs (#1–2) as well as on those near the northeastern edge of the study site (reefs #8–10). Their abundance decreased slightly but significantly with levels of snorkeling (Fig 7). Two other species of sea urchins (purple urchins *Arbacia punctulata* and slatepencil urchins *Eucidaris tribuloides*) were observed; both of the latter species grouped together occurred at abundances of 0–2 individuals m^-2^. They exhibited the opposite trend to that of *D*. *antillarum*, in that their grouped abundance increased slightly but significantly with snorkeling level (Fig 7); trends for all sea urchins remained significant when analyses included only the inner bay sites (Table 2).

## Discussion

This study reveals high rates of recreational snorkeling on some patch reefs at Akumal Bay, Mexico. Live stony coral cover decreased significantly and macroalgal cover increased significantly with increased levels of snorkeling visitation on reefs in this bay. The intensive snorkeling activity in some areas of the bay (> 1,000 in-water visitors d^-1^) thus may negatively impact many of the stony corals, potentially opening reef space for algal colonization and leading to these reefs becoming macroalgal-dominated, as known for some other coral reefs exposed to human pressure [52,59]. In-water visitation during peak use hours of the day approximately doubled over a 2 y period in high-use sections of this bay, from ~170 snorkelers at 15:00 in 2013 (High Use zone; CEA; [17]) to ~ 340 snorkelers at 16:00 in 2015 (sections I and J, present study). Even more rapid growth occurred in the once low-use sections of this bay, of ~ 400% increase in 2 y with ~ 14 snorkelers at 15:00 in 2013 (Low Use zone; CEA; [17]) to 57 at 16:00 in 2015 (sections G and H, present study). Further increases in abundances of snorkelers in the bay were observed during subsequent observations in 2016 (B. Renfro, pers. obs.; CEA). This bay has begun to receive high levels of in-water visitation only in recent years [17,60,61], which may explain in part why many types of reef organisms, including sea anemones, symbiotic crustaceans [46], and stony corals (present study) remain abundant on some reefs in this bay. The geomorphology of Akumal Bay also likely creates conditions that cause variation in benthic composition among the patch reefs [43]. If the trend of growing snorkeler pressure is reversed soon and managed at sustainable levels [18], both the living coral reefs in this bay, and the tourism industry that depends on them, potentially can be preserved.

Recent completion of a beach resort hotel along the mid-bay likely contributed to expansion of the once clearly high- versus low-use sections [12], into a very high use section at the northeast end, a moderate and growing-use mid section, and a shrinking section of low use in the southwest of this bay. This expansion occurred despite designation by CEA of a U-shaped snorkeling route in the northeast bay (sections I and J in Fig 1), designed to spatially concentrate human-reef contact and thus relieve pressure on patch reefs in the remainder of the bay, thereby aligning future visitor use with the previously-documented high and low use areas [12]. The designated snorkeler route often was not followed in 2015 due to lack of enforcement (B. Renfro, pers. obs.). Our results confirm that intensive snorkeling activity expanded between 2013 and 2015 into reefs of the mid bay (section H, Fig 1), and that as of 2015, live stony coral cover was low not only in sections J and I but also in H, relative to more extensive stony coral in the southern bay where snorkelers had not yet become abundant. The observed shift in community structure from stony corals to dominance by macroalgae likely contributes to reduced biodiversity, because macroalgae inhibit stony coral settlement and subsequent reef accretion, limiting the formation of complex coral reef habitats that support diverse organisms [62,63]. In contrast, coral reef sea anemones and their crustacean associates remain relatively abundant in section H, indicating that they may be more resilient to intensive reef visitation than are stony corals, on at least a short-term basis [46].

The species-specific trends in stony coral cover observed here may have been caused by several factors. The relatively high percent cover of mounding coral *Orbicella annularis* on some patch reefs was unexpected, given the decline of this species recently throughout the Caribbean [64–66]. However, colonies of *O*. *annularis* alter their reproductive strategies to asexually reproduce more in areas of high disturbance [67], which potentially could boost the percent cover of this species where divers disturb the benthos. In the U.S. Virgin Islands, colonies of *O*. *annularis* recently have exhibited similar trends of increased but localized recruitment, supporting the idea that this species could be resilient and recovering in some regions of the Caribbean [68]. Small branching corals *Porites porites* also occurred on patch reefs with high snorkeling levels, despite the tendency of their skeletons to fracture when kicked or bumped by visitors [16]. Colonies of *P*. *porites* dominate some disturbed coral reefs [69], and are considered to be weedy corals with rapid growth that may permit them to quickly colonize space opened by the decline of other stony coral species, allowing this stony coral to become a “winner” on rapidly changing reefscapes [66,70]. Colonies of elkhorn coral *Acropora palmata* were once abundant at Akumal, as evidenced by their large branching skeletal remains on both the reef crest and deeper portions of the inner bay (B. Renfro, pers. obs.). Both *A*. *palmata* and staghorn corals *A*. *cervicornis* historically dominated reefs throughout the Caribbean Sea [71], but have declined drastically due to the compounding influences of anthropogenic and natural disturbances [72,73], including hurricanes Emily and Wilma which both hit the Yucatan Peninsula in 2005 [48,49]. The relationships examined here between the remaining acroporid percent cover and snorkeling level disappeared when the outer bay (reef crest) sites were removed, indicating their percent cover may be driven more by outer-inner bay physical gradients than snorkeler pressure. Colonies of *Agaricia tenuifolia* are weedy corals which colonize open space rapidly and may take over reefs after acroporids die off [74–76]. However, breakage of the fragile, leafy plates of *A*. *tenuifolia* can outpace rapid skeletal growth [74,77]. Colonies of the symmetrical brain coral *Diploria strigosa* have been predicted to dominate Caribbean reefs as “winners” in the face of growing anthropogenic disturbance [70,78]. Their larval settlement is impaired by the presence of macroalgae [79], so the high algal cover observed here in heavily-visited bay sections could deter *D*. *strigosa* recruitment. Whatever the causes, our data on high variability among stony coral types in their relationships with snorkeling intensity on these reefs suggest that reef-builders may exhibit species-level complexity in their responses to human contact.

The patterns documented here in the cover of both turf algae and erect calcified macroalgae (CMA) are similar to those reported from other coral reefs exposed to intense human activity [80–82], and in particular to in-water visitation [17]. Macroalgae may expand in percent cover when reef space opens for colonization following the decline of competing stony corals [31,83], and also when they are released from top-down control due to the overharvest [5,6] or disturbance [29] of herbivorous fishes. The presence of snorkelers leads to an immediate reduction in the percent time that herbivorous fishes spend foraging on macroalgae in Akumal Bay [29], possibly acting as a mechanism driving some of the percent cover trends in algae reported here. Conversely, crustose coralline algae (CCA), which serves as important substrate for juvenile stony coral settlement, exhibited trends similar to those observed in other reef systems [80,81]. In-water visitors increase sedimentation on coral reefs by disturbing adjacent soft substrates [84], and may reduce CCA through this mechanism, because the percent cover of CCA decreases with reef sediment load [85]. The variation observed here in fleshy macroalgal percent cover was unexpected, given that it typically increases with human disturbance on coral reefs as part of a shift to overall macroalgal dominance [81,86]. Further investigation is needed to determine factors impacting the abundance of fleshy macroalgae in this bay. We conclude that complex factors interacting with the biology of each reef species may synergistically contribute to the variation observed here among some of the benthic taxa, in their percent cover patterns relative to levels of recreational snorkeling. Effects of indirect, multi-level ecological interactions need be taken into account in future studies that examine community trends in relation to visitation on coral reefs.

The abundance of long-spined sea urchins *Diadema antillarum* was highly variable within some of the examined reef sites, so it is not possible to make firm conclusions about impacts of snorkeler activity. These sea urchins were once major herbivores on reefs in the Caribbean, together with some fishes [31,87]. During the 1980s they experienced a mass die off due to disease [87], and are now slowly recovering in some areas [33,88] including Mexico [57]. The relatively high observed abundance of *D*. *antillarum* urchins on the outer reef crest could be due in part to the presence of large *A*. *palmata* skeletons that provide more protective crevice habitat than available at inner bay sites, potentially exacerbated by snorkeler breakage of stony corals in the inner bay. In contrast, abundances of the 2 other sea urchin species exhibited patterns which could relate to their small body size and robust, short spines, making them relatively insensitive to disturbance by snorkelers. The latter urchins also likely require less complex habitat (smaller reef holes) for protection than do *D*. *antillarum*, allowing them to inhabit the relatively low-relief reefs of the inner bay. Studies are needed to further assess the potential effects of recreational snorkeling and related disturbances on sea urchins.

The trends observed here for fire corals, gorgonians and zoanthids (zoantharians) indicate that these 3 types of reef cnidarians could be sensitive to reef disturbances caused by intensive snorkeling activity. Some studies have reported that gorgonians are not much affected by anthropogenic disturbances [56,89], while others have quantified negative diver impacts [90]. Some fire corals possess delicate, easily-fractured skeletons and respond to disturbance similarly to delicate branching stony corals (scleractinians) [91], in that they are easily broken by divers [84,90]. Mechanisms causing the observed trend for zoanthids (zoantharians) are not clear; as a group, zoanthids do not exhibit consistent trends in relation to anthropogenic disturbances [92–94]. Identification of members of these groups to the genus or species level in future studies would allow clearer conclusions to be drawn about their trends in relation to anthropogenic impacts.

Other factors in addition to recreational snorkeling pressure potentially contributed to the trends in the benthic communities recorded here. The inner and outer bay sites differed slightly in depth below sea level (~ 1.5 m depth difference) and distance from shore (~ 120–200 m vs. ~ 250–300 m distant, respectively), but even when outer bay sites were excluded from analyses, significant trends remained for many of the benthic organisms examined. Freshwater seeps in Akumal Bay also release nutrients from polluted groundwater in the adjacent watershed [95], leading to stony coral growth inhibition and death [96]. However, freshwater seeps at Akumal occur mostly in the mid to south bay where snorkeling is infrequent, resulting in elevated nutrients in those areas relative to the north bay [43] where snorkeling activity is most intense. Dissolved nutrient levels vary along the Quintana Roo coast of the Yucatan Peninsula [47], and also fluctuate among years with levels of tourist visitation [97]. Local variation within Akumal Bay in levels of nutrient pollution and elevated sea temperatures both exhibit north-south spatial patterns opposite to that of snorkeling levels within the bay [43], so neither can fully explain the benthic community patterns reported here. The north end of Akumal Bay also contains boating lanes and mooring zones whose effects may impact nearby patch reefs. Boat noise disrupts the settlement behavior of larval coral reef fishes [98], and the chemicals and heavy metals used in antifouling paints and boat cleaning agents can leach into the surrounding water, causing damage to adjacent reef ecosystems [99]. Boat traffic and facilities in the north bay thus may disturb reef organisms and act synergistically with snorkeling to impact the reef benthos. Much of the boat and automobile traffic at the north end the bay brings in recreational snorkelers and thus is linked spatially to the intensive snorkeling activity ([17] B. Renfro pers. obs.). Chemical and noise pollution from these transportation sources are unlikely to be the sole agents responsible for the coral reef patterns reported here, because some of the benthic reef damage was observed in the form of stony coral skeletal fracture and tissue abrasion, both known to be inflicted by snorkelers [16,24].

We conclude that the most intensively-visited coral reefs in Akumal Bay probably have exceeded their carrying capacity for recreational snorkeling, and that they exhibit low stony coral and high macroalgal cover relative to other parts of the bay. Levels of visitation in intensively-used section J (> 1,000 snorkelers day^-1^, up to ~ 300,000 year^-1^) were 50 times higher than the carrying capacity estimated for coral reefs of ~ 5000–6000 guided dives site^-1^ year^-1^ [84]. Government limitation of recreational use to the snorkeling paths set up by CEA could prevent the spread of this reef degradation, and allow areas outside the snorkeling zones to recover. Additional management actions could include the local hotels encouraging guests to participate in guided dives, and the restriction of snorkel gear rental to individuals entering the water with a guide. Both of these management actions would support the education and employment of local residents as trained snorkel guides. Tour groups led by educated guides who conduct pre-dive briefings and manage visitor behaviors in-water cause significantly less damage to reefs than less well-managed groups [16]. The employment of local snorkeling guides would both limit negative impacts to the environment as well as boost the local economy [18]. Akumal residents who work as in-water guides appear to be passionate about their reef resources and more committed to regulating snorkeler behavior than are the non-local guides who bring in daily busloads of snorkelers from Cancun (B. Renfro, pers. obs.). Due to the presence of important sea turtle and coral reef resources in Akumal Bay, in 2016 the Mexican government decreed this area as a refuge for protected marine species; then in 2017, the bay temporarily was closed to in-water snorkeling activities [100]. If this governmental action is accompanied by the enforcement of sustainable management practices, these coral reefs may survive the massive incursion of tourist activity that appears to be extending from Cancun southwards along the Yucatan coastline.

Effective carrying capacities for recreational snorkeling on coral reefs can be reached through various means, such as spatially limiting human-reef contact to mapped snorkel tour routes, utilizing in-water guides who provide pre-dive educational briefings as well as intervene when snorkelers exhibit reef-damaging behaviors, and limitation of the total number of snorkelers visiting each reef site. Regular scientifically-based monitoring of the reef benthos and fish communities subsequently can be used to support and direct adjustments to these management efforts. The patterns of recreational snorkeling and reef community structure described here have implications for other coral reef areas exposed to intensive visitation. They reveal that benthic reef organisms may exhibit complex, species-specific variation with in-water snorkeling pressure, and that targeted monitoring of key species is needed to provide scientifically-based support for the management of heavily-visited reefs.

## Supporting information

S1 Table **Raw data on (a) visitation rates within examined sections of Akumal Bay, Mexico, (b) percent cover of benthic organisms, and (c) abundance of sea urchins on coral reefs surveyed in the bay.** See map Fig 1 and text for details.
(XLSX)
